# A new era for *Journal of Biological Research-Thessaloniki*

**DOI:** 10.1186/2241-5793-21-1

**Published:** 2014-05-13

**Authors:** Theodore J Abatzopoulos

**Affiliations:** Department of Genetics, Development and Molecular Biology, School of Biology, Aristotle University of Thessaloniki, 54124 Thessaloniki, Greece

*Journal of Biological Research-Thessaloniki* (*JBR*) replaced the Scientific Annals of the School of Biology of the Aristotle University of Thessaloniki called *BIOS* in 2004. In these 10 years, more than 850 authors originating from 54 countries have published 280 articles. *JBR* has been indexed in ISI Journal Citation Reports^©^ (Thomson Reuters), since 2008 (current Impact Factor 0.62). Until the end of 2013, *JBR* was published by the Aristotle University of Thessaloniki. With the rapidly changing environment of scientific publishing, it was decided that *JBR* should be transformed into a modern, open access journal. For this transition, the Editors of *JBR* along with the Administration of the Aristotle University of Thessaloniki decided to become partners with one of the world leaders in open access publishing, BioMed Central. Starting from May 2014 (Volume 21), *JBR* articles will be published by BioMed Central and freely available online at http://www.jbiolres.com/ [1]. All the back volumes of *JBR* (Volumes 1–20) are also freely available at http://www.jbr.gr/ [2], which will remain active for archiving purposes.

Dissemination of scientific results is the milestone of science and the vehicle for transporting knowledge produced in universities and other research institutes to the community. Open access publishing ensures high visibility of content, gives authors copyright of their work, and articles are indexed in freely accessible full-text repositories, in compliance with the policies of numerous funding organizations/bodies [3–7]. Also, open access fulfills the USA demand for government-funded research papers to be made freely available within one year of publication [5] and the requirement posed by the Wellcome Trust and Research Councils UK for government-funded research results to be published open access [6, 7]. For all the above reasons, we were enthusiastic to move *JBR* to publish under a “gold” open access model, which provides immediate open access to all articles published in the journal.

Another issue that the Editors of *JBR* consider of great importance is research and publication ethics. This includes data fabrication/falsification/misinterpretation, disclosing of relevant personal or financial interests, plagiarism, and protection of sensitive and confidential data. BioMed Central is a member of, and subscribes to the principles of, COPE (Committee on Publication Ethics) [8], and provides Editors with access to CrossCheck software for plagiarism detection. In addition, BioMed Central assures the fast publication of accepted manuscripts in its final citation, and also gives authors the opportunity to publish large datasets, video and large numbers of color illustrations for no additional charge [9].

*JBR* carries the valuable (and variable) lessons learned during its first ten years of life over to a fresh new start brimming with new challenges and responsibilities, as well as opportunities. With an enriched set of article types (Research, Reviews, Short Reports, Hypotheses, Letters to the Editor, Commentaries and Book Reviews), the journal now provides additional podia through which authors may communicate their research and ideas in more flexible and exciting ways. Reflecting the broad expertise of the Editorial Board and the philosophy of its Editors, *JBR* continues on the same track of a multidisciplinary scope within biology. We hope that the broad coverage also incites authors to submit interesting works of an interdisciplinary/integrative nature. Within this general biological scope, the Editors of the journal will publish series devoted to popular topics such as evolutionary biology and molecular ecology.

*JBR* is run by practicing scientists, who are recognized academics that continue to publish and serve as reviewers for other journals. Therefore, authors and *JBR* staff (across all levels of responsibility, from the Editor-in-Chief to the Editorial Board) share common interests. Neither subsidiaries nor any scientific society endorse *JBR*. Within this structure the question of novelty in scientific research is fundamental. The criteria of novelty have been hotly debated over the years [10], more so nowadays as some feel that the open access publishing model threatens to redefine them. *JBR* does not intend to be defiant of nor enticed by the metric of Impact Factor. As scientists judge scientists, it is useful to keep in mind that scientific progress is mainly incremental and occasionally interrupted by leaps of novelty.

*JBR* now enters a new exciting period of wider exposure and rigorous assessment. The Editors of *JBR* intend to follow the same principles that guided them to date in keeping on a tangent line with scientific progress and ethics of publishing.

## References

[1] *Journal of Biological Research-Thessaloniki*. http://www.jbiolres.com/

[2] *Journal of Biological Research-Thessaloniki (previous website)*. http://www.jbr.gr/

[3] **Which funding agencies explicitly allow direct use of their grants to cover article processing charges?** http://www.biomedcentral.com/about/apcfaq

[4] **NIH Calls on Scientists to Speed Public Release of Research Publications**. http://www.nih.gov/news/pr/feb2005/od-03.htm

[5] Van Noorder R (2013). US science to be open to all. Nature.

[6] **Wellcome Trust position statement in support of open and unrestricted access to published research**. http://www.wellcome.ac.uk/node3302.html

[7] RCUK Policy on Open Access. http://www.rcuk.ac.uk/research/outputs/

[8] Committee on Publication Ethics. http://publicationethics.org/

[9] *Why publish your article in Journal of Biological Researc-Thessaloniki?*. http://www.jbiolres.com/about#whypublish

[10] Arnqvist G (2013). Editorial rejects? Novelty, schnovelty!. Trends in Ecol & Evol.

